# WHO's Investigations and Studies, Unity Studies: A global initiative creating equitable opportunities for enhanced surveillance, operational research, capacity building, and global knowledge sharing

**DOI:** 10.1111/irv.13256

**Published:** 2024-02-12

**Authors:** Isabel Bergeri, Nicki L. Boddington, Hannah C. Lewis, Lorenzo Subissi, Sophie von Dobschuetz, Angel Rodriguez, Jorge Jara, Hala Abou El Naja, Amal Barakat, Arash Rashidian, Eman Abdelkreem Aly, Lubna Al Ariqi, Pushpa Wijesinghe, Francis Inbanathan, Phuong Nam Nguyen, Manilay Phengxay, Linh‐Vi Le, Temuulen Enebish, Joseph Okeibunor, Belinda Herring, Elise Farley, Pernille Jorgensen, Aisling Marie Vaughan, Joshua Mott, Wenqing Zhang, Richard Pebody, Maria D. Van Kerkhove

**Affiliations:** ^1^ WHO Health Emergencies Programme, World Health Organization Headquarters Geneva Switzerland; ^2^ Pan American Health Organization Washington District of Columbia USA; ^3^ World Health Organization for the Eastern Mediterranean Cairo Egypt; ^4^ Regional Office for South‐East Asia, World Health Organization New Delhi India; ^5^ Regional Office for the Western Pacific, World Health Organization Manila Philippines; ^6^ Regional Office for Africa, World Health Organization Brazzaville Republic of the Congo; ^7^ Regional Office for Europe, World Health Organization Copenhagen Denmark

**Keywords:** COVID‐19, emerging respiratory pathogens, influenza, pandemic preparedness

## Abstract

The World Health Organization's Unity Studies global initiative provides a generic preparedness and readiness framework for conducting detailed investigations and epidemiological studies critical for the early and ongoing assessment of emerging respiratory pathogens of pandemic potential. During the COVID‐19 pandemic, the initiative produced standardized investigation protocols and supported Member States to generate robust and comparable data to inform public health decision making. The subsequent iteration of the initiative is being implemented to develop revised and new investigation protocols, implementation toolkits and work to build a sustainable global network of sites, enabling the global community to be better prepared for the next emerging respiratory pathogen with epidemic or pandemic potential.

## 1 BACKGROUND AND HISTORY OF WHO'S INVESTIGATIONS AND STUDIES, “UNITY STUDIES”

In the early stages of the emergence of a novel influenza virus or other emerging respiratory pathogen with epidemic or pandemic potential, rapidly gathering and analyzing epidemiological, virological, and clinical data, including infection‐severity, transmissibility, and other infectious disease parameters, are critical to rapidly assess the situation and to guide public health decision making and response actions. The World Health Organization's (WHO) Investigations and Studies, also known as the “Unity Studies” global initiative,^1^ provides a generic preparedness and readiness framework for conducting targeted investigations and studies. These investigations and studies are critical tools to supplement routine surveillance systems to address specific public health objectives, particularly in the early stages of a pandemic, but also over the course of the event through a continuous, periodic (e.g., to assess and inform the use of interventions, such as vaccines), or alert driven (e.g., emergence of a new variant or lineage) assessment process. The initiative developed from work carried out since the 2009 influenza pandemic, which has been previously described by Bergeri et al.^2^

## 2 UNITY STUDIES DURING THE COVID‐19 PANDEMIC

The declaration of a public health emergency of international concern due to the novel coronavirus, severe acute respiratory syndrome coronavirus 2 (SARS‐CoV‐2), responsible for the coronavirus disease 2019 (COVID‐19) on January 30, 2020, prompted rapid implementation of early investigations to inform appropriate national and global public health actions. The suite of existing pandemic preparedness early investigation protocols was rapidly adapted for SARS‐CoV‐2 in the first weeks after the detection of the novel coronavirus with the First Few X (FFX) cases and contacts transmission and household transmission protocols made available on the WHO webpage by January 30, 2020.^3^ These were developed under the WHO initiative, Unity Studies, and promoted globally for the implementation of high‐quality, standardized investigations.^2^, ^3^ Ten protocols using eight study methodologies were ultimately developed and their global uptake has been previously described by Bergeri et al.^2^

Implementation was supported (technical support, provision of serological assays, funding, and training) by WHO and its partners globally, with emphasis also to support building surveillance and operational research capacities in low‐ and middle‐income countries (LMICs). For example, the initiative delivered sero‐assays to 53 member states (MS) across all six WHO regions, with the majority (over 90%) going to LMICs.

The three levels of the WHO (HQ, Regional offices [ROs], and country offices [COs]) worked in collaboration with technical partners to implement this work through existing mechanisms. The WHO HQ Unity Team convened regular coordination calls with key partners such as United States Centers for Disease Control, Pasteur Institute, and Bill and Melinda Gates Foundation to share information, optimize resources, and encourage harmonization between partners and alignment with government priorities.

During the pandemic, the initiative was responsive to the needs of MS study groups and, in addition to the above‐mentioned protocols, developed additional guidance for study groups. This included “Adapting Unity Studies protocols to COVID‐19 vaccine rollout,” Interim Technical Notes on “Adapting First Few X cases and contacts (FFX) and Household Transmission (HHTI) Investigation study protocols to COVID‐19 variants,” and an operational brief for “Interpreting SARS‐CoV‐2 seroprevalence studies for public health decision‐making” (available on request from unity@who.int).

Primarily, the results of the WHO Unity Studies were used nationally and sub‐nationally by MS to inform public health decision making. Furthermore, the global adoption and the early sharing of results with WHO helped generate robust and comparable data to inform regional and global public health decision making and actions.^4^, ^5^, ^6^, ^7^, ^8^ Although ownership of primary data remained firmly with sites and MS, early results were shared directly with WHO for timely assessment and uploaded to an online repository, Zenodo,^9^ by study teams. Aggregated results shared with WHO facilitated several pooled analyses including a number of systematic reviews and meta‐analyses that were undertaken with support from technical partners such as the University of Melbourne and SeroTracker.^5^, ^6^, ^7^ Global SARS‐CoV‐2 seroprevalence estimates generated by the Unity Studies have been considered by the WHO Strategic Advisory Group of Experts on Immunization in their review “the emerging evidence on the characteristics and potential benefits of hybrid immunity”^10^ and their recent recommendations on prioritizing the use of COVID‐19 vaccines.^11^

The Unity Study initiative also supported capacity building in LMIC across all regions. In addition to direct technical support on study design, protocol development, and statistical analyses to investigators, several scientific country support workshops (such as scientific writing, data management, and data analysis) were held across the regions and in different United Nations languages, to build broader research capacities and to support investigators to publish their work in peer‐reviewed scientific journals. To further support countries in sharing findings and improving equity in publication, WHO has supported this Special Issue of COVID‐19 Unity Studies with Influenza and Other Respiratory Viruses (IORV). WHO has provided financial support to authors allowing them to access writing and editing support provided by Wiley, prior to submission of their articles, as well as funding the publication fees for accepted articles. The collection features investigations standardized to three of the WHO's Unity Studies protocols: (1) population‐based age‐stratified sero‐epidemiological investigation protocol, (2) FFX cases and contact investigations, and (3) household transmission investigations. The collection, focused exclusively on LMIC, aims to reduce barriers commonly experienced by authors from countries that receive less funding and support, and by doing so the Special Issue hopes to encourage submissions from all regions of the world and reduce publication bias to high‐income countries. It also aims to highlight the importance of standardized epidemiological studies to improve global comparisons, guide emergency public health decision making, and strengthen future pandemic readiness efforts.

## 3 FUTURE OF INVESTIGATIONS AND STUDIES, UNITY STUDIES 2.0

**FIGURE 1:**
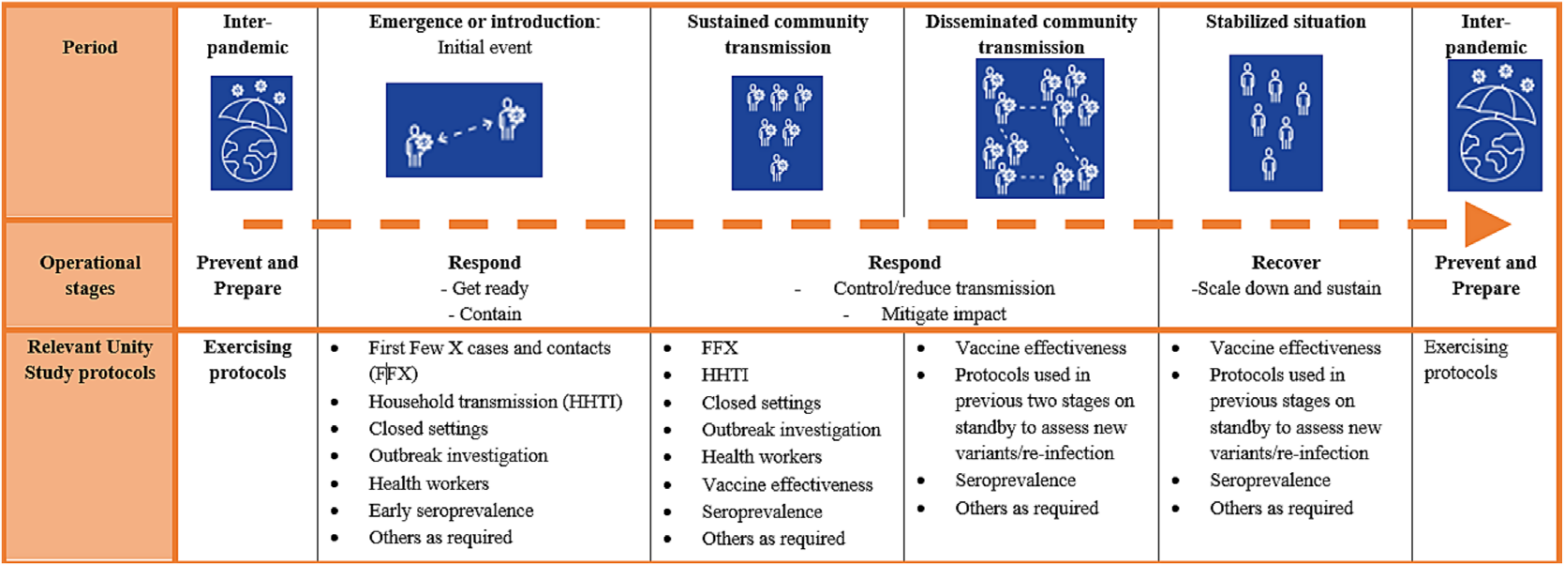
Unity studies protocols aligned with the pandemic periods of the organizing framework for respiratory pathogen pandemic planning, preparedness, and resilience for emerging threats (PRET).^13^

**FIGURE 2:**
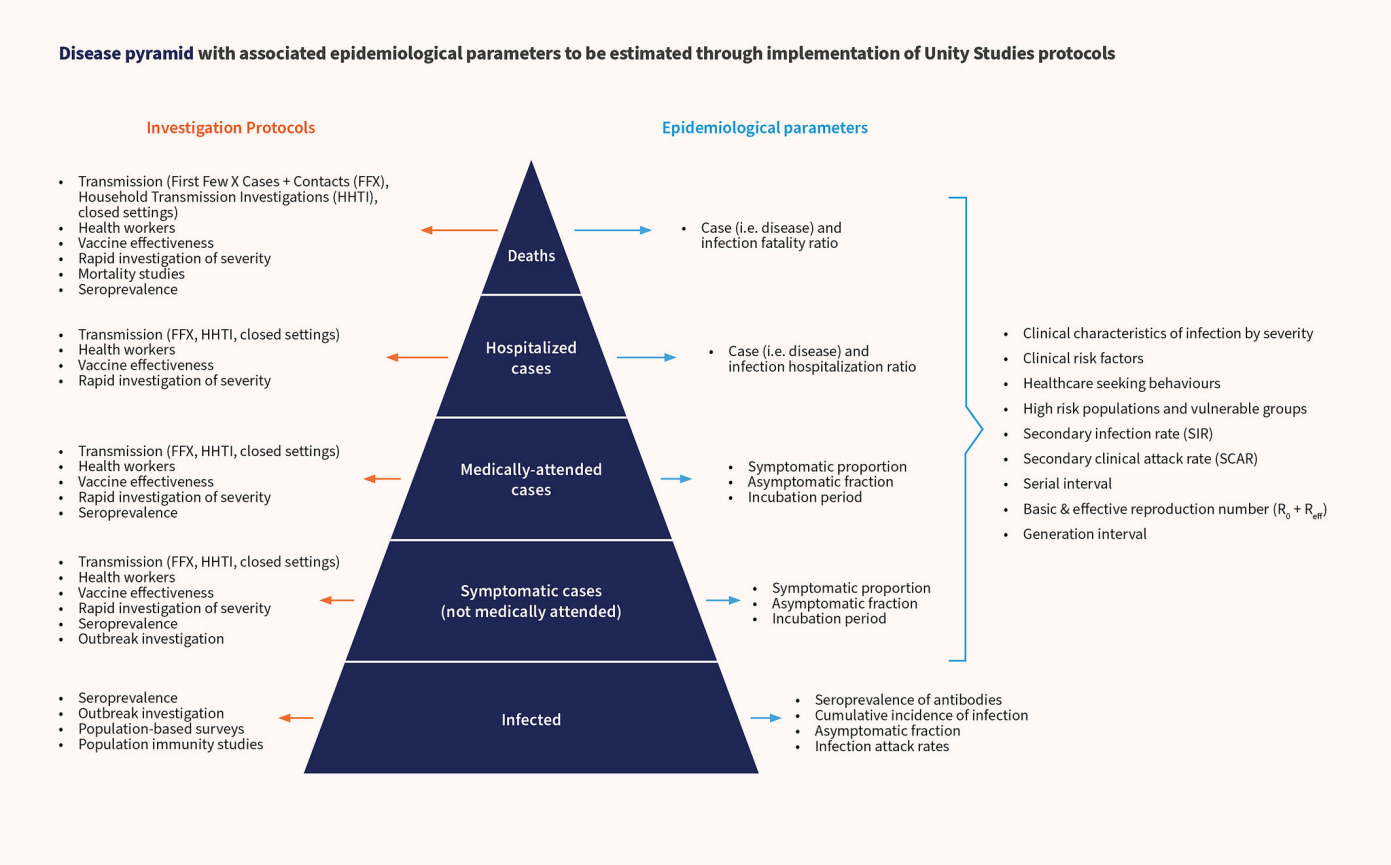
Disease pyramid with associated epidemiological parameters to be estimated through implementation of Unity Studies protocols.

As we move toward an interpandemic period, countries must develop sustainable and resilient integrated surveillance strategies to monitor influenza, SARS‐CoV‐2, and other emerging respiratory pathogens with epidemic or pandemic potential. As it is not feasible to address the complex needs of respiratory virus surveillance with a single system, multiple fit‐for‐purpose surveillance approaches and complementary investigations must fit together as tiles in a “mosaic.”^12^ Within this mosaic, discrete studies and investigations such as the Unity Studies can address certain public health objectives that are not efficiently met by other existing systems, such as rapidly assessing transmissibility, estimating population susceptibility/immunity and infection severity, aiding the identification of high‐risk population groups for targeted interventions, and estimating the burden of disease, vaccine effectiveness, and severity.^12^ Given this context and building on the lessons learned during the COVID‐19 pandemic, WHO is planning the Unity Studies 2.0 initiative, to ensure operational readiness for future pandemic. This is an “at the ready” international framework for preparedness and response to future pandemics. It will address both disease‐specific aspects (such as for pandemic influenza and coronaviruses) and be applicable to any emerging respiratory pathogen with epidemic or pandemic potential. As part of this, standardized protocols, both disease‐specific and generic to emerging respiratory pathogens with epidemic or pandemic potential, are being revised and developed. To date, a number of protocols have been published including the transmission and severity protocols (First Few X cases and contacts [FFX], household transmission investigation [HHTI], and closed setting investigation) for influenza as well as for respiratory pathogens with pandemic potential.^1^, ^13^ Figure 1 shows the different protocols and where they might be useful in answering key public health questions along the pandemic preparedness continuum according to the organizing framework for respiratory pathogen pandemic planning, Preparedness and Resilience for Emerging Threats (PRET) guidance.^1^, ^14^ The standardized protocols can be used to estimate many epidemiological parameters across each level of the disease pyramid (Figure 2), noting that these protocols will not address all domains of pandemic surveillance or operational research and will work in complement with other surveillance approaches.^12^ Additional protocols covering areas such as assessing early severity, zoonotic/One Health, public health and social measures (PHSM), and social and behavioral science are also underway.

Work is also underway to develop a global operational network of sites that are primed to conduct country‐specific standardized, pre‐planned, and pre‐approved investigations and studies. Network sites will normally be national institutes or national academic institutions (e.g., universities). Each site will have one main focal point, which will be responsible for fulfilling the Terms of Reference of the network and may coordinate other study sites within the country.^1^ The investigations and studies will be exercised through “peace time” such as during seasonal influenza epidemics. The network will comprise representative settings, building on countries who successfully implemented Unity Studies protocols during the COVID‐19 pandemic as well as those supported by the High‐Level Implementation Plan, Pandemic Influenza Preparedness (PIP) Partnership Contribution (PC),^15^ and thus including settings with available capacity or through collaborations to develop local capacity where it is otherwise limited.

The Unity Studies initiative will be supported through the development and use of implementation toolkits such as data analysis plans, as well as the development of an acceptable architecture for a timely results repository. Key focus areas of this collaborative and globally coordinated initiative will be equity, capacity building, country ownership, and improving science translation with the aim of improving the ease of results sharing with decision makers. Furthermore, the initiative aims to be flexible with a view to being able to respond to disease X.

In summary, the Unity Studies initiative provided valuable contributions during the COVID‐19 pandemic to aid epidemiological understanding and risk assessment, guiding the use of interventions such as public health and social measures and vaccines. The initiative created equitable opportunities for enhanced surveillance and capacity building and established a connected community of implementing partners across countries. This Special Issue showcases studies conducted in LMIC demonstrating the feasibility of conducting and usefulness of the Unity Studies in these settings and has facilitated global knowledge sharing and a collaborative spirit, which are key values that will be taken forward into the Unity Studies 2.0 initiative.

## AUTHOR CONTRIBUTIONS

**Isabel Bergeri:** Conceptualization; methodology; project administration; writing—original draft; writing—review and editing. **Nicki L. Boddington:** Project administration; writing—original draft; writing—review and editing. **Hannah C. Lewis:** Project administration; writing— original draft; writing—review and editing. **Lorenzo Subissi:** Data curation; project administration; writing—review and editing. **Sophie von Dobschuetz:** Writing—review and editing. **Angel Rodriguez:** Data curation; investigation; writing—review and editing. **Jorge Jara:** Data curation; investigation; writing—review and editing. **Hala Abou El Naja:** Data curation; investigation; writing—review and editing. **Amal Barakat:** Data curation; investigation; writing—review and editing. **Arash Rashidian:** Data curation; investigation; writing—review and editing. **Eman Abdelkreem Aly:** Data curation; investigation; writing—review and editing. **Lubna Al Ariqi:** Data curation; investigation; writing— review and editing. **Pushpa Wijesinghe:** Data curation; investigation; writing—review and editing. **Francis Inbanathan:** Data curation; investigation; writing—review and editing. **Phuong Nam Nguyen:** Data curation; investigation; writing—review and editing. **Manilay Phengxay:** Data curation; investigation; writing—review and editing. **Linh‐Vi Le:** Data curation; investigation; writing—review and editing. **Temuulen Enebish:** Data curation; investigation; writing—review and editing. **Joseph Okeibunor:** Data curation; investigation; writing—review and editing. **Belinda Herring:** Data curation; investigation; writing—review and editing. **Elise Farley:** Data curation; investigation; writing—review and editing. **Pernille Jorgensen:** Data curation; investigation; writing—review and editing. **Aisling Marie Vaughan:** Data curation; investigation; writing —review and editing. **Joshua Mott:** Writing—review and editing. **Wenqing Zhang:** Conceptualization; writing—review and editing. **Richard Pebody:** Conceptualization; writing—review and editing. **Maria D. Van Kerkhove:** Conceptualization; writing—review and editing.

## CONFLICT OF INTEREST STATEMENT

No conflict of interest was declared.

### PEER REVIEW

The peer review history for this article is available at https://www.webofscience.com/api/gateway/wos/peer-review/10.1111/irv.13256.

## Data Availability

Data sharing is not applicable; no new data are generated.
